# Effects of a Very-Low-Calorie Ketogenic Diet on the Fecal and Urinary Volatilome in an Obese Patient Cohort: A Preliminary Investigation

**DOI:** 10.3390/nu15173752

**Published:** 2023-08-28

**Authors:** Giuseppe Celano, Francesco Maria Calabrese, Giuseppe Riezzo, Benedetta D’Attoma, Antonia Ignazzi, Martina Di Chito, Annamaria Sila, Sara De Nucci, Roberta Rinaldi, Michele Linsalata, Mirco Vacca, Carmen Aurora Apa, Maria De Angelis, Gianluigi Giannelli, Giovanni De Pergola, Francesco Russo

**Affiliations:** 1 Department of Soil, Plant and Food Science, University of Bari Aldo Moro, 70126 Bari, Italy; giuseppe.celano@uniba.it (G.C.); mirco.vacca@uniba.it (M.V.); carmen.apa@uniba.it (C.A.A.); maria.deangelis@uniba.it (M.D.A.); 2 Functional Gastrointestinal Disorders Research Group, National Institute of Gastroenterology IRCCS “S. de Bellis”, 70013 Castellana Grotte, Italy; giuseppe.riezzo@irccsdebellis.it (G.R.); benedetta.dattoma@irccsdebellis.it (B.D.); antonia.ignazzi@irccsdebellis.it (A.I.); michele.linsalata@irccsdebellis.it (M.L.); 3 Center of Nutrition for the Research and the Care of Obesity and Metabolic Diseases, National Institute of Gastroenterology IRCCS “S. de Bellis”, 70013 Castellana Grotte, Italy; dichitomartina@gmail.com (M.D.C.); annamaria.sila@irccsdebellis.it (A.S.); sara.denucci@irccsdebellis.it (S.D.N.); roberta.rinaldi@irccsdebellis.it (R.R.); giovanni.depergola@irccsdebellis.it (G.D.P.); 4 Scientific Direction, National Institute of Gastroenterology IRCCS “S. de Bellis”, 70013 Castellana Grotte, Italy; gianluigi.giannelli@irccsdebellis.it

**Keywords:** dysbiosis, inflammation, intestinal barrier, ketogenic diet, metabolome, obesity, short-chain fatty acids, volatile organic compounds

## Abstract

Several recent studies deepened the strong connection between gut microbiota and obesity. The effectiveness of the very-low-calorie ketogenic diet (VLCKD) has been measured in terms of positive impact on the host homeostasis, but little is known of the modification exerted on the intestinal metabolome. To inspect this complex relationship, we analyzed both fecal and urinary metabolome in terms of volatile organic compounds (VOCs) by the GC-MS method in 25 obese patients that were under VLCKD for eight weeks. Partial least square discriminant analysis evidenced specific urinary and fecal metabolites whose profile can be considered a signature of a partial restore toward the host eubiosis. Specifically, among various keystone VOCs, the decreased concentration of four statistically significant fecal esters (i.e., propanoic acid pentyl ester, butanoic acid hexyl ester, butanoic acid pentyl ester, and pentanoic acid butyl ester) supports the positive effect of VLCKD treatment. Our pilot study results suggest a potential positive effect of VLCKD intervention affecting fecal and urinary volatilome profiles from obese patients. Meta-omics techniques including the study of genes and transcripts will help in developing new interventions useful in preventing or treating obesity and its associated health problems.

## 1. Introduction

Suffering from complex and heterogenous metabolic symptoms, obese patients experience a wide range of health problems. Recent research studied the role of the intestinal metabolome in obese patients [1]. The intestinal metabolome is inclusive of a panel of small molecules produced by the gut microbiota that significantly affect the host metabolism [2]. Compared to lean controls, obese individuals proved to harbor a different gut microbiota taxa composition together with altered relative abundances; this profile has also been reflected by metabolome intestinal modifications [3]. Focusing on low-grade inflammatory pathologies, Thingholm et al. [4] pushed towards the need of identifying the existing associations between obesity, type 2 diabetes, the gut microbiome, and the plasma metabolome. A systematic review of human studies characterizing gut-microbiota-related metabolites in obesity stated how gut-microbiota-related low-molecular-weight metabolites were significantly up- or down-regulated in overweight and obese individuals [5]. Additionally, a recent paper described lower abundance in specific taxa related to the glutamate-fermenting pathways, i.e., Bacteroides thetaiotaomicron decreased in obese individuals versus lean controls [6].

Excessive caloric intake and specific foods can lead to an altered balance of the intestinal microbiota and to epithelial damage too [7]. The second condition is associated, in turn, with an increased intestinal permeability and translocation of luminal content versus the underlying mucosa [8]. Morbidly obese patients show impairment of the intestinal barrier functions, together with an increased inflammatory response in the systemic compartment [9]. Downstream to all of this reported evidence, the gut microbiota is able to: (i) Increase energy production from food, (ii) Provide a low-grade inflammation status, and (iii) Impact the fatty acid tissue composition. All these three listed factors are favorable to the crosslink between the gut microbiota and obesity. However, in contrast with the evidence of this recent literature, some works support the absence of a direct link between obesity and metabolic disorders [10].

From a therapeutic point of view, data indicated how important weight loss interventions can improve metabolism and inflammation status in severely obese individuals who were candidates for bariatric surgery. At the same time, a significantly altered fecal metabolome was monitored in these patients [11].

In the last few years, due to its effectiveness in determining weight loss, the ketogenic diet (KD) became much more popular, and even the more restrictive VLCKD, boosted by its safe therapeutic intervention, was readily used by people suffering from obesity [12,13]. VLCKD has proved to have beneficial effects on body composition, metabolic profile, inflammation, and oxidative stress gene expression [14,15]. Additionally, regardless of keeping under consideration concomitant factors counteracting NAFLD disease progression, VLCKD leads to metabolic reduction in insulin levels, a lowering of insulin resistance (IR), the reduction of body weight/fat mass, and the induction of ketosis. This evidence supports the diet’s efficacy and allowed for considering this therapeutic strategy as a helpful tool [16].

Since VLCKD is a very low-carbohydrate diet that induces a state of ketosis, it may have important implications on the intestinal barrier function, the gut microbiome, and its derived products. Recently, our group conducted preliminary research on the effects due to VLCKD on the intestinal permeability of patients with obesity [17]. Results confirmed significant beneficial changes after VLCKD in anthropometric and biochemical features but, notably, also highlighted a significant increase in intestinal permeability and markers of dysbiosis, specifically in a subgroup of patients in which the intestinal barrier functions significantly worsened at the end of treatment. As a matter of fact, to our knowledge, there is little information on the effects of a VLCKD on the fecal metabolome of obese patients.

An imbalance in the relative abundance of Bacteroidetes and Firmicutes was initially linked to the development of obesity through its effect on host energy balance, IR, and inflammation [18]. However, recent evidence suggests a more complex modulation than a mere shift in this ratio where other factors may play a crucial role [19]. In this connection, the exact mechanisms underlying the relationship between the fecal metabolome and obesity are still not fully understood. Host factors, including genetics and lifestyle, may contribute to shaping the fecal metabolome and its association with obesity. Zierer et al. [20] analyzed a wide spectrum of more than one thousand metabolites in a large individual cohort from a twin population study (TwinsUK) and stated how fecal metabolome was poorly influenced by host genetics but much more strongly associated with visceral fat mass. Importantly, this suggests how potential mechanisms from microbiota may impact abdominal obesity.

Other authors have investigated the relationship between weight loss interventions, such as diet and bariatric surgery, and fecal metabolome, with inconsistent findings. Salazar et al. [21] described a singular correlation between Firmicutes and an excess of body weight, as derived by measuring the body mass index. In any case, the low-calorie diet showed little impact of taxa composition and related metabolic activity.

As for the VLCKD, different studies performed in humans [22,23] and in vivo [24], mainly addressed to investigating the effects of KD in neurological dysfunctions, have shown that this diet can alter the abundance and diversity of gut bacteria [25]. These changes can lead to an altered production of metabolites by the gut microbiota, which can be detected in the fecal metabolome.

By inspecting the gut microbiota composition and its relative metabolites, we may be able to develop new interventions useful in preventing or treating obesity and its comorbidities. Based on these premises, to face this issue, the urinary and fecal samples from 25 obese patients who underwent eight weeks of VLCKD were analyzed for their metabolome profiles before and after the diet.

## 2. Materials and Methods

### 2.1. Study Design and Population

This pilot study was performed at the Italian Center of Nutrition for the Research and the Care of Obesity and the Metabolic Diseases afferent to the National Institute of Gastroenterology IRCCS “Saverio de Bellis”, Castellana Grotte (Ba).

Obese patients, ages ranging from 18 to 65 years with a BMI greater than 30 kg/m^2^, underwent medical history, physical examination, and laboratory tests. A total of 25 patients who met the above listed inclusion criteria and that did not have irritable bowel syndrome (IBS) were selected.

While collecting their medical history, participants were asked to report their smoking and daily alcohol consumption habits and, more precisely, based on the American and European guidelines, if they consumed more than two (or one in the case of female) glasses of alcohol per day [26,27]. The threshold for men was set at 30 g/day, while that for women was 20 g/day.

The exclusion criteria included participants that had hypersensitivity to components contained in meal replacement products, type 1 diabetes mellitus, history of cerebrovascular and cardiac diseases, respiratory insufficiency, severe GI diseases, chronic kidney disease with an estimated glomerular filtration rate lower than 60, psychiatric issues, pregnancy, and lactation [9,10,11,12]. Moreover, eating disorders, serious mental illnesses, liver failure, substance abuse, frail elderly patients, active/severe infections, and rare diseases, and disorders of mitochondrial fatty acid oxidation were considered conditions in the exclusion criteria. Additionally, participants were not allowed to take drugs, probiotics, vitamins, or supplements and were required to discontinue these supplements 15 days before starting the diet.

The internal Medical Ethical Committee approved the study protocol (Prot. n. 170/CE De Bellis), and the study was conducted in accordance with the Helsinki Declaration of 1964. Written consent was obtained from each participant before their involvement in the study. This study was registered at ClinicalTrials.gov with the following identifier: NCT05477212.

### 2.2. Study Design

**Figure 1:**
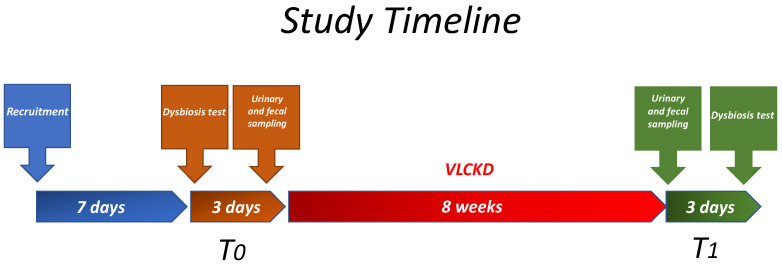
Timeline treatment and gathering steps.

The study recruited patients with obesity between April and November 2022. The initial screening foresaw the administering of the Gastrointestinal Symptom Rating Scale (GSRS) questionnaire during the recruitment visit. Follow-up visits were scheduled in two medical appointments: one before the diet (T_0_) and another eight weeks after the diet (T_1_), as shown in Figure 1. Anthropometric measurements and data related to fasting blood, urine, and fecal samples were collected at T_0_ and T_1_. Patients were asked to come back within 2–4 days for blood sampling, anthropometric measurements, and assessment of intestinal dysbiosis. After completing the entry tests, the patients began their personalized VLCKD, and blood was withdrawn a second time at the end of the diet program. Lastly, within three days following the diet program’s completion, patients underwent dysbiosis tests again.

### 2.3. Diet Protocol

In this present research, the authors used a previously published protocol, whose first two steps are based on those described by Bruci et al. [14]. All participants followed a VLCKD plan based on a two-step protocol from New Penta, Cuneo, Italy. The carbohydrate intake was fixed at 20–50 g per day, whereas the protein intake was fixed at 1–1.4 g/kg of ideal body weight. Finally, the lipid intake was fixed at 15–30 g per day. Participants were advised to drink a minimum of 2 L of water daily, and the daily calorie intake was lower than 800 Kcal. Micronutrient supplements were provided during the entire dietary treatment as recommended to prevent nutritional deficits. In the first step, only meal replacements with specific quantities and types of vegetables were allowed, while a protein dish was included in replacement of one of the substituted meals in the following second step.

### 2.4. Anthropometric Parameters and Biochemical Characteristics

The researchers measured the participants’ body mass index (BMI), which is the ratio of body weight to height squared, by measuring their weight and height while fasting, and with an empty bladder. Taken measurements were obtained using a calibrated scale and stadiometer. Blood samples were collected early in the morning (8:00 and 9:00 a.m.) after a fasting phase lasting the whole night. Standard laboratory methods were used to determine the participants’ biochemical features before and after they followed the very-low-calorie ketogenic diet. The semi-quantitative concentration of acetoacetic acid was measured by patients who collected the first morning’s urine themselves before the start of the treatment and each week until the end of the study (Ketur-Test, Accu-Chec, Roche Diagnostics, Monza, Italy). All the patients showed the presence of ketosis in the urine samples collected during the diet. Since we used a semi-quantitative method, this finding would confirm that ketogenesis was present in almost all the patients, allowing us to define our diet as ketogenic.

### 2.5. Fecal Metabolome

Obese patient fecal samples were used to evaluate the volatile organic compounds (VOCs) profile before and after VLCKD diet. The micro-extraction procedure described by Calabrese et al. [28] was slightly modified in order to obtain the best extraction efficiency. A 1 gram quantity of fecal samples with 10 μL of 4-methyl-2-pentanol (final concentration 9.9 μg/g) was placed into 10 mL glass vials and sealed with polytetrafluoroethylene (PTFE)-coated silicone rubber septa and then equilibrated for 10 min at 60 °C. To extract volatile compounds by CombiPAL system injector autosampler (CTC Analytics, Zwingen, Switzerland), a conditioned 50/30 µm DVB/CAR/PDMS fiber (Supelco, Bellefonte, PA, USA) was exposed to headspace for 50 min at 60 °C. The injection was carried out by transferring the fiber into the heated injection port (220 °C) in splitless mode, and helium was used as the carrier gas at a flow rate of 1 mL/min. For the GC-MS analysis, a Clarus 680 (Perkin Elmer, Beaconsfield, UK) gas chromatography equipped with an Rtx-WAX column (30 m × 0.25 mm i.d., 0.25 µm film thickness) (Restek, Bellefonte, PA, USA) was used and coupled to a Clarus SQ8MS (Perkin Elmer) with source and transfer line temperatures kept at 250 and 230 °C, respectively. The oven temperature was initially set at 35 °C for 8 min, then increased to 60 °C at 4 °C/min, to 160 °C at 6 °C/min, and finally to 200 °C at 20 °C/min and held for 15 min. Electron ionization masses were recorded at 70 eV in the mass-to-charge ratio interval, *m*/*z* 34 to 350. The generated chromatograms were analyzed for peak identification using the National Institute of Standard and Technology 2008 (NIST) library. A peak area threshold of >1,000,000 and 85% or greater match probability was used for VOCs identification, followed by manual visual inspection of the fragment patterns when required. 4-Methyl-2-pentanol (final concentration 33 mg/L) was used as an internal standard in all analyses to quantify the identified compounds by interpolating the relative areas versus the standard internal area.

### 2.6. Quantitative Analysis of Targeted VOCs in Fecal Samples

A targeted analysis of short-chain fatty acids (SCFAs) and branched-chain fatty acids (BCFAs) was conducted to evaluate the differences between groups before and after treatment. Acetic acid, butyric acid, propionic acid, isobutyric acid, and isovaleric acid (Sigma-Aldrich, St. Louis, MO, USA) and internal standard IS (final concentration of 1 mg/L) were quantified by using a standard curve. Selective ion monitoring (SIM) mode was used to evaluate the concentration of each compound [29]. The peak area of SCFAs in the fecal sample was integrated, and the absolute concentration (ppm) of SCFAs in the fecal sample was calculated by using the calibration curve equation including the response value relative to IS.

### 2.7. Indican and Skatole Evaluation

Morning urine samples were collected from all patients and analyzed using a standard colorimetric assay kit (Indican Assay Kit, ABNova Corporation, Taipei, Taiwan) to detect and measure urinary indican and skatole levels. The analysis was performed using a Thermo Scientific model Dionex high-performance liquid chromatography (HPLC) system with the 3-methylindole kit (EurekaLab Division, Chiaravalle, AN, Italy), as previously reported [30]. According to previous research [31], urinary levels of indican and skatole above 20 mg/L and 20 µg/L, respectively, are considered indicators of fermentative or putrefactive dysbiosis.

### 2.8. Statistical Analysis

This study represents a pilot investigation, as no previous research has examined the effects of very-low-carbohydrate ketogenic diets on the intestinal metabolome. Therefore, the researchers did not perform a statistical power calculation due to the study’s exploratory nature.

Partial least square differential analysis (PLS-DA) was computed by means of the PLSR.Anal function in MetaboAnalystR-software (version 2.0.0), run in the R-environment. When appropriate, a paired Wilcoxon test was used to compare groups based on time stratification. All discussed results are relative to statistically significant variables, obtained after running a multiple test correction. Correlation analysis (Pearson’s correlation coefficients) was run by using the R “corr.test” function (https://search.r-project.org/CRAN/refmans/correlation/html/cor_test.html, accessed on 8 June 2023) and graphically rendered by using the R “corrplot” (https://cran.r-project.org/web/packages/corrplot/index.html) package, accessed on 8 June 2023. Only statistically significant correlations were plotted.

## 3. Results

The backbone of our analysis workflow relies on fecal and urine metabolomics datasets. Both these VOC datasets were inspected in terms of statistically significant variables by means of statistical test comparison, helpful in disclosing the similarity of patient samples before and after the VLCKD administration. As previously stated in our obese patient cohort, the VLCKD determined a statistically significant reduction in BMI values, together with a decrease in FT3, glucose, gamma GT, and ALT. Moreover, dealing with the lipidic profile, reductions in total cholesterol, triglycerides, HDL-, LDL-, and total cholesterol triglycerides were detected [17]. Included participants had a BMI > 30. In greater detail, 14 subjects (56%) were in the range of BMI from 30–35, 6 individuals (24%) were in the BMI range 35–40, and the remaining 5 subjects (20%) had a BMI > 40.

### 3.1. Untargeted Analyses (GC-MS) of VOCs in Fecal Samples before and after the VLCKD Administration

**Figure 2:**
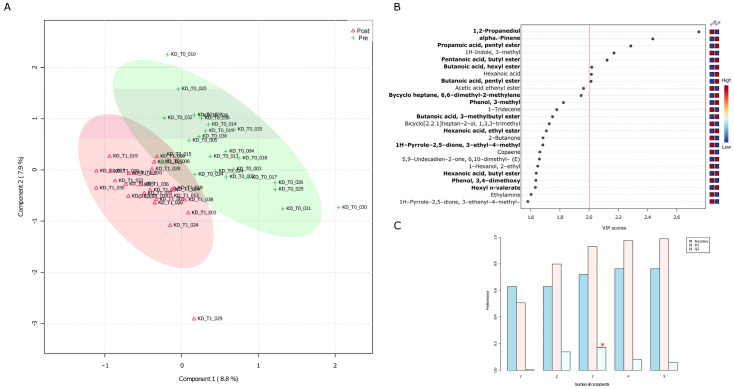
PLS-DA of fecal VOCs based on pre- and post- group belonging. (**A**) PLS-DA plot. Fecal VOC normalized abundance matrix was used as input to compute the PLS-DA. T scores were used to plot samples onto a bidimensional plot. Green and red ellipses are relative to pre- and post-VLCKD administration, respectively. (**B**) Most contributing variables, based on the “scores of variable importance on projection” (VIP scores), are those over threshold. Based on the color legend, higher and lower contributions were marked as red and blue squares, respectively. (**C**) For each component a cross-validation analysis was used to estimate Q2, R2, and the accuracy of the PLS model that works independently of the specific subset used as training set when Q2 value tends to R2. The asterisk indicates the goodness of the component accuracy.

When the normalized (range scaled) matrix of fecal metabolomics was inspected through a partial least squared discriminant analysis (PLS-DA), pre- and post-administered samples appeared to be distributed into two almost distinct clouds (Figure 2A). The top twenty-five “Variable Importance in Projection” (VIP) VOCs had scores included in the range 1.5–2.8 and their impact on sample stratification was supported by cross validating coefficients. Q2 coefficient (Figure 2C), which tends to R2, indicates the goodness of PLS-DA relatively to the third principal component as measured by means of a permutation test between components. Ten fecal VOCs were marked by VIP scores higher than 2, and mostly contributed to sample stratification. In other words, the PLS model works independently of the specific data used to train the model.

The over threshold VOC set precisely included 1,2-propanediol and 1H-Indole, 3-methyl- (skatole) that increased after patients were administered with the VLCKD diet (red squares in the right-side color diagram), whereas alpha. -pinene, propanoic acid pentyl ester, pentanoic acid butyl ester, butanoic acid hexyl ester, hexanoic acid, butanoic acid pentyl ester, and (1S)- decreased in their concentrations. Except for hexanoic acid and skatole, the other seven over threshold VOCs also emerged as statistically significant results from the Wilcoxon rank sum test (Supplementary Table S1).

### 3.2. Untargeted Analyses (GC-MS) of VOCs in Urine Samples before and after the VLCKD Administration

Non-invasive GC-MS method was used to retrieve the set of markers that constitute the urine volatilome.

**Figure 3:**
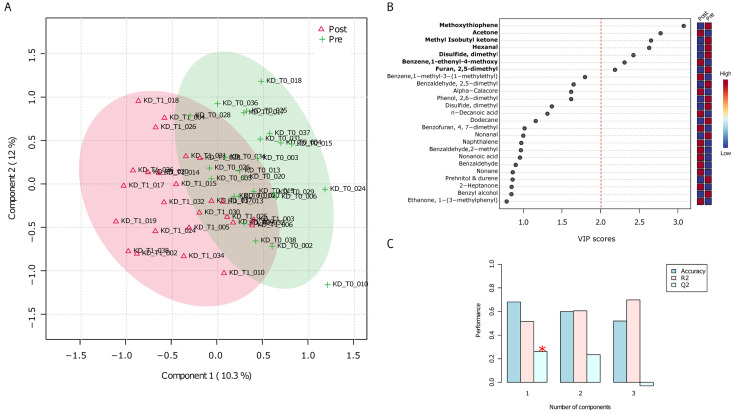
PLS-DA of urine VOCs based on pre- and post- group belonging. (**A**) PLS-DA plot. Urine VOC normalized abundance matrix was used as input to compute the PLS-DA. T scores were used to plot samples onto a bidimensional plot. Green and red ellipses are relative to pre- and post-VLCKD administration, respectively. (**B**) Most contributing variables, based on the “scores of variable importance on projection” (VIP scores), are those over threshold. Based on the color legend, higher and lower contributions were marked as red and blue squares, respectively. A score of 2 was chosen to select most impacting variables. (**C**) For each component a cross-validation analysis was used to estimate Q2, R2, and the accuracy of the PLS model that works independently of the specific subset used as training set when Q2 value tends to R2. The asterisk indicates the goodness of the component accuracy.

The normalized (range scaled) whole matrix of urine metabolomics was given as input for a partial least squares discriminant analysis (PLS-DA). Pre- and post-administered samples appeared to be distributed into two partially overlapping clouds (Figure 3A). The top twenty-five “Variable Importance in Projection” (VIP) VOCs had scores included in the range 0.8–3.1 (Figure 3B) and their impact on sample stratification was supported by cross validating coefficients Q2/R2X/R2Y (Figure 3C). A significative and high value of Q2, which tends to R2, indicates the goodness of PLS-DA and it has been measured by means of a permutation test between components. Seven VOCs were marked by VIP scores higher than 2, indicating how they mostly contributed to the cloud separation. In other words, the PLS model works independently of the specific data used to train the model.

The volatile over threshold group precisely included 2-methoxythiophene, methyl isobutyl ketone, hexanal, disulfide dimethyl, and furan, 2,5-dimethyl- that decreased their concentration in those patients after being under the administered VLCKD diet (blue squares in the color diagram); whereas benzene, 1-ethenyl-4-methoxy-, and acetone increased.

Urine VOC with high VIP score (>2) from PLS-DA also emerged as statistically significative variables in a two-group Wilcoxon rank sum test run from VLCKD-T0 and VLCKD-T1 (Supplementary Table S2).

### 3.3. SCFAs Profile

**Figure 4:**
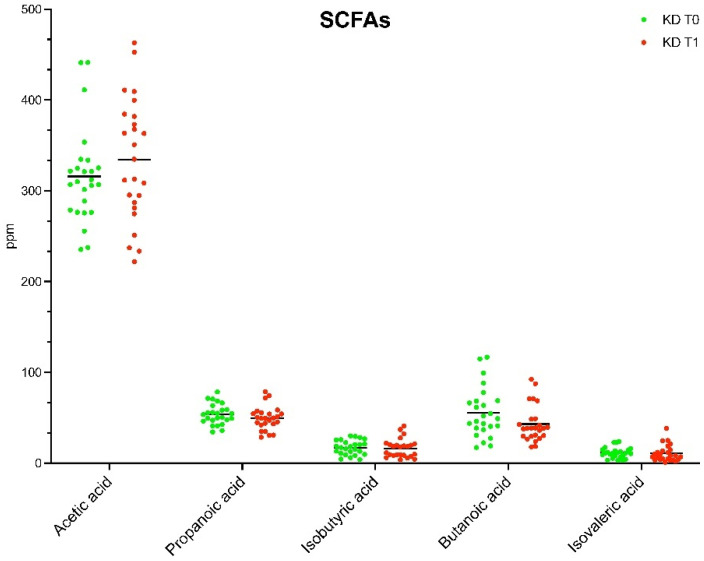
Concentrations (ppm) of short-chain fatty acids (SCFAs) in headspace fecal samples of patients with obesity before (T_0_) and after (T_1_) eight weeks of VLCKD. After applying the Wilcoxon matched pairs signed rank test, only butanoic acid was significantly decreased at T_1_.

A targeted GC-MS experiment was used to inspect the short chain fatty acid (SCFA) profiles. Differences between fecal SCFAs in patients with obesity before and after 8 weeks of VLCKD administration were investigated by means of a paired Wilcoxon sum rank test. The targeted analysis of fecal samples highlighted the content in ppm of the main SCFAs (Figure 4). The only significant difference was found in the level of butanoic acid (Table 1), which was significantly lower in T_1_ than in T_0_ (*p* = 0.048).

### 3.4. Correlation Analysis between BMI and Volatilome Variables

BMI was significantly lowered as a consequence of the administration of VLCKD (Supplementary Figure S1).

To assess the relationship between obesity indices and volatilome metabolites, we used a Pearson’s correlation test between BMI and VOCs that statistically differed between T_0_ and T_1_. As a result, BMI positively correlated (*p* < 0.05) with hexanal, p-aminotoluene, alpha-pinene, ciclopentadecane, phenol, 3-methyl-5-(1-methylethyl)-, methylcarbamate dimethyl-disulfide, and 2,6-dimethyl- Phenol (Supplementary Figure S2 and Supplementary Table S3).

### 3.5. Intestinal Dysbiosis

At baseline, the urinary indican concentrations in the patients with obesity were approximately twice the level of 20 mg/L (42.72 ± 4.12 mg/L), suggesting the presence of fermentative dysbiosis. At the end of the diet, a significant (*p* = 0.006) increase in urinary concentrations occurred (57.40 ± 3.38 mg/L) (Supplementary Figure S3A). The urinary skatole concentrations were below the level of 20 µg/L, and they were unaffected by diet (4.28 ± 0.41 µg/L vs. 4.84 ± 0.38 µg/L; Pre diet vs. Post diet, *p* = 0.282) (Supplementary Figure S3B).

## 4. Discussion

Obesity is a complex metabolic disorder characterized by the accumulation of excessive body fat, leading to various related pathologies such as diabetes, hypertension, cardiovascular diseases, and cancer. Several recent studies deepened the link between obesity and gut microbiome, a complex ecosystem of microorganisms residing in the human GI tract [32]. Very-low-calorie ketogenic diets (VLCKD) are widely employed as successful weight-loss strategies in obese patients [16]. Different diets impact the metataxonomic composition of gut microbiota, and relative associated metabolic pathways [33].

Also, alongside the type of diet, the length of dietary interventions has been shown to deeply modify the microbiota consortium composition and interactions [34].

To inspect this complex relationship, the fecal volatilome proves handy for the identification of those metabolites produced by microorganisms or derived by specific host metabolism and that significantly impact the host’s physiology [35].

At the same time, here we used the gas chromatography coupled with mass spectrometry (GC-MS) method on fecal and urinary metabolome of obese patients before and after eight weeks of VLCKD administration. The urine specimen metabolites serve as a powerful tool to discover potential biomarkers linked to obesity.

Multivariate statistical analysis methods evidenced how specific metabolites resulting from targeted and untargeted volatilomics were altered due to diet intervention. We thus deepened the indication derived from our previous published result on urine, revealing an increase in indole (also named as 3-indoxyl sulfate) and 3-methyl-indole (or skatole), derivatives of the tryptophan catabolism, both markers of altered intestinal permeability [17], and that were in turn associated with an altered microbial gut metabolism [36]. Simultaneous and higher levels of both these molecules are suggestive of a fermentative/putrefactive dysbiosis of the small intestine and colon. Our untargeted fecal GC-MS experiment was concordant with our previous data and evidenced significant increased levels of skatole in fecal samples. This finding was not confirmed by the evaluation of urine volatilome profiles, most likely due to GC-MS limit of detection (ca. 1 ppm).

As recently reported by Zgarbová and Vrzal, skatole has a two-coin effect whose benefits easily shift, in case of increased concentrations, to toxicity. Its increased levels prompt its role as a marker of intestinal disease development and at the same time it has been hypothesized that the molecule induces pulmonotoxicity [37].

Our PLS-DA multivariate analysis identified, among the top fecal ranked VIP scores, few VOCs that increased after the VLCKD administration. The highest VIP score was relative to 1,2-propanediol, a microbial fermentation product derived from the hydrolyzation of dietary fibers, as well as from fucosylated human milk oligosaccarides, or host mucins, such as rhamnose and fucose [38]. In this respect, the capacity to produce propionate or butyrate from hexose sugars relies on different taxa, and particularly on some species belonging to the Lachnospiraceae family that are known for their capacity to switch from butyrate to propionate metabolism depending on the substrate [39]. Gut commensal bacteria shape their interaction based on cross-feeding that depends on lactate, acetate, and succinate, but also on 1,2-propanediol; all important intermediary metabolites for the production of short-chain fatty acids (SCFA) [40]. 1,2-propanediol is part of the metabolism of thiosulfate oxidation and, together with ethanolamine, can proceed using tetrathionate intermediate as an electron acceptor in place of molecular oxygen [41]. Under normal gut conditions, the oxidation of thiosulfate to tetrathionate is minimal, but results in being accelerated by inflammation processes when the gut increases its oxidative potential [41]. A sub-chronic inflammation is typically associated with obesity and diabetes [42].

In summary, as a result of the prompted trophic interaction sustained by 1,2-propanediol, ecologically stable metabolic networks are implied in the production of SCFA, recognized keystone elements in host physiology [43]. Notably, our targeted GC-MS experimental results revealed how an increased (not significant) concentration of acetic acid marked samples at T1, whereas a decrease in all the other tested SCFA emerged. The only statistically significant value found was for the butanoic acid. This SCFA is the major source for the trophic activity of the intestinal epithelium, and its decrease seems to counteract health-promoting effects of VLCKD [44].

A progressive decrease in butyrate producer bacteria taxa, i.e., *E. rectale*, *Roseburia*, and *F. prausnitzii* has been monitored during the shift from normal to medium- and very-low-carbohydrate intake diets [45]. The two above mentioned species are included in the set of commensal taxa known to exert a key activity in the production of SCFA, and mainly of butyrate. The high reduction in carbohydrate intake determined a strong impact on the diversity and richness of gut microbiota [46] and, advisedly, leads to an impairment in microbiota byproduct availability. In line with this evidence, we found low levels of butyrate after VLCKD administration [47].

Among fecal decreased VOC after 8 weeks of the diet, alpha pinene, belonging to the polyphenolic group of terpene, known for its hypolipidemic and anti-obesity effects [48], was positively correlated with BMI. Our data are in line with results from a recent paper by Deledda et al. [49] reporting a strong and negative correlation of ketogenic diet with the pinene degradation pathway. Having a hyperglycemic effect, pinene reduction can be a consequence of blood glucose reduction or can be a direct consequence of VLCKD.

This finding was also in line with, and supported by, the reduction in glucose levels in our patient cohort [17].

As further evidence, we noticed an increased production of esters and carboxylic acid derivative compounds in obese patients after treatment. Their presence could be referred to oxidative stress, which promotes lipid peroxidation downstream. More precisely, the production of alcohols and carboxylic acids in the presence of oxygen-reactive species (radical-mediated lipid peroxidation) leads to esters formation [50].

The decreased concentrations of four statistically significant esters we observed in fecal samples at T1 (i.e., propanoic acid pentyl ester, pentanoic acid butyl ester, butanoic acid hexyl ester, and butanoic acid pentyl ester) are indicative of a restoration of physiologic and metabolic changes occurring during the weight gain in obese patients.

Another fecal VOC with lowered concentration at T1 is the hexanoic acid, a medium chain fatty acid (MCFA) whose production is enhanced in livers of obese mice with insulin resistance [51]. At the same time, obese men showed higher hexanoic and butyrate postprandial levels in triglycerides [52]. Interestingly, hexanoic acid was shown to protect against dysbiosis and to prevent the expansion of pathogenic bacteria [53]. In humans, fecal levels of this MCFA were inversely correlated with Crohn’s disease activity and significantly decreased in the fecal samples of patients with inflammatory bowel diseases (IBDs) [54]. Moreover, the increase in this metabolite has been evaluated in fructose intolerant patients after probiotic treatment, with an improvement of clinical symptoms (e.g., bloating, abdominal pain) [55].

The urinary peak areas of benzene, 1-ethenyl-4-methoxy- (belonging to the chemical class of anisole), and acetone increased at T_1_. The anisole has been previously detected at statistically significant higher levels in a cohort of patients suffering from colorectal cancer when compared with lymphoma and leukaemia patients [56]. The other increased urinary VOC was acetone, the least abundant among the ketone bodies that can be expelled through urine, and breath because of its high vapor pressure (breath acetone has also been weighted as an indicator of ketosis in adults under ketogenic diet [57].)

Dealing with significantly decreased VOCs, our analyses detected 2-methoxythiophene, methyl isobutyl ketone, hexanal, dimethyl disulfide, and furan, 2,5-dimethyl-. Moreover, hexanal and dimethyl disulphide decreased in association with BMI (*p* < 0.05), as shown in our correlation analysis.

It has been suggested that a significant enhancement in aldehydes is related to lung cancer patients compared to healthy controls [58] as well as increased 2-methoxythiophene and dimethyl disulfide levels in breast cancer patients [59]. Increased contents of sulphur-containing metabolites, namely 2-methoxythiophene and dimethyl disulphide, were detected as statistically significant VOCs in leukaemia patients in comparison with normal controls [56]. Among ketogenic diet markers, the aldehyde hexanal, a derivative of lipid peroxidation and a decomposition product of linoleic acid, has also been recognized to be a marker of oxidative stress [50]. The hexanal decrease could be associated with the antioxidant and anti-inflammatory effect exerted by ketogenic diet [60].

Furans and alkylfurans can derive from various precursors in food, like ascorbic acid, UFA, amino acids, and carotenoids [61] and their presence, detected in urine samples, was ascertained to derive from food products, including coffee and canned food [62]. As a result of thermal processing, furan and its derivatives are known for their capacity to induce oxidative stress [63]. Thus, the reduced concentration found after diet administration can be considered as a marker indicating the reduction in oxidative-related metabolisms.

## 5. Conclusions

The relationship between obesity and the fecal metabolome is complex and multifaceted. Present data and recent studies from our group and others have shown that alterations in the fecal metabolome and the gut lumen’s overall state of health are associated with obesity and metabolic disorders and can be modulated by weight loss interventions. Although some metabolites need to be evaluated in a more complex cohort, and a metagenomics experiment would be helpful in elucidating the contribution of specific taxa in the production of microbial VOCs, the GC-MS VOC panel analyzed here supports the safety and effectiveness of the VLCKD dietary regimen in reducing body weight. At the same time, this diet significantly modifies the fecal and urinary metabolome. Some markers linked to cancer and to oxidative stress resulted in being reduced, whereas other important metabolites with trophic activity towards colon epithelium eubiosis decreased (i.e., butanoic acid). All these findings would suggest a contradictory effect of VLKCD in terms of antioxidant activity coupled with an alteration of permeability.

As a limitation of the study, only a semi-quantitative method was used to quantify ketone bodies in urine during the dietary intervention. No evaluation of ketone body concentration concerned blood samples. Nonetheless, all patients exhibited ketosis in urine, which made us confident in considering our diet as ketogenic. The usage of more effective detection methods will improve our confidence in future studies.

Further research is needed to fully understand the underlying mechanisms and develop targeted interventions to modulate the fecal metabolome to improve metabolic health in obese individuals.

## Figures and Tables

**Table 1:** Wilcoxon sum rank test of SCFA from targeted GC-MS in obese patients before (T_0_) and after (T_1_) the VLCKD administration. Average plus/minus standard deviations have been reported for each SCFA, together with *p*-values (*p*).

SCFAs (ppm)	T_0_	T_1_	*p*
Acetic acid	315.87 ± 52.67	334.59 ± 67.17	0.278365
Propanoic acid	53.79 ± 0.32	49.43 ± 12.82	0.247017
Isobutyric acid	17.07 ± 0.94	16.32 ± 10.14	0.786834
Butanoic acid	55.51 ± 27.43	42.96 ± 20.10	0.048502
Isovaleric acid	11.46 ± 5.95	10.69 ± 8.91	0.749362

## Data Availability

The datasets used and/or analyzed during the current study are available from the corresponding author upon reasonable request.

## Supplementary Materials

The following supporting information can be downloaded at https://www.mdpi.com/article/10.3390/nu15173752/s1, Table S1: Wilcoxon rank sum test on fecal VOCs before and after the VLCKD; Table S2: Wilcoxon rank sum test on urine VOCs before and after the VLCKD; Table S3: Pearson’s correlation analysis between BMI and VOCs; Figure S1: BMI spaghetti graph. The spaghetti graph shows the reduction in BMI after VLCKD treatment (q < 0.05); Figure S2: Pearson’s correlation between BMI and significant VOCs. BMI values and fecal/urinary metabolites were correlated by means of the R “corr.mtest” function and graphically rendered by using the R “corrplot package”. Only statistically significant correlations were plotted. The color scaled bar indicates positive and negative correlations as green and purple circles, respectively. BMI relative correlations were indicated by aqua-colored background squares; Figure S3: Urinary indican (panel A) and urinary skatole (panel B) levels in patients with obesity before (T_0_) and after (T_1_) eight weeks of VLCKD. Data expressed as means ± SEM. Wilcoxon matched-pairs signed-rank test was used to compare pre-treatment and post-treatment data. Differences were considered significant at *p* < 0.05. The dotted line indicates the cut-off level for dysbiosis: indican (20 mg/L) and skatole (20 µg/L).
