# Supplementary material for: Effects of a Very-Low-Calorie Ketogenic Diet on the Fecal and Urinary Volatilome in an Obese Patient Cohort: A Preliminary Investigation
Source: Nutrients. 2023 Aug 28;15(17):3752. doi: 10.3390/nu15173752 (PMC10490432; doi:10.3390/nu15173752)
Supplement: Supplementary file 1 [file nutrients-15-03752-s001.zip › nutrients-2548704-supplementary.pdf]

Supplementary figures and tables

Supplementary Figure S1. BMI spaghetti graph. The spaghetti graph shows the reduction of BMI after VLCKD treatment ($q < 0.05$).

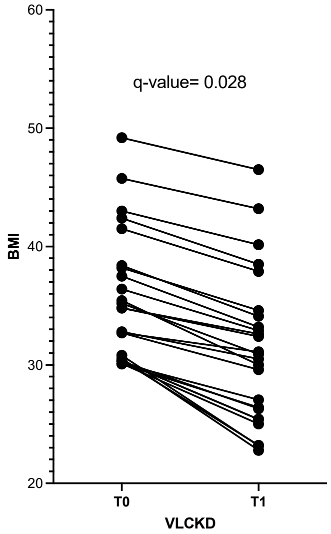

Supplementary Figure S2. Pearson's correlation between BMI and significant VOCs. BMI values and fecal/urinary metabolites were correlated by means of the R "corr.mtest" function and graphically rendered by using the R "corrplot package. Only statistically significant correlations were plotted. The color scaled bar indicates positive and negative correlations as green and purple circles, respectively. BMI relative correlations were indicated by aqua colored background squares.

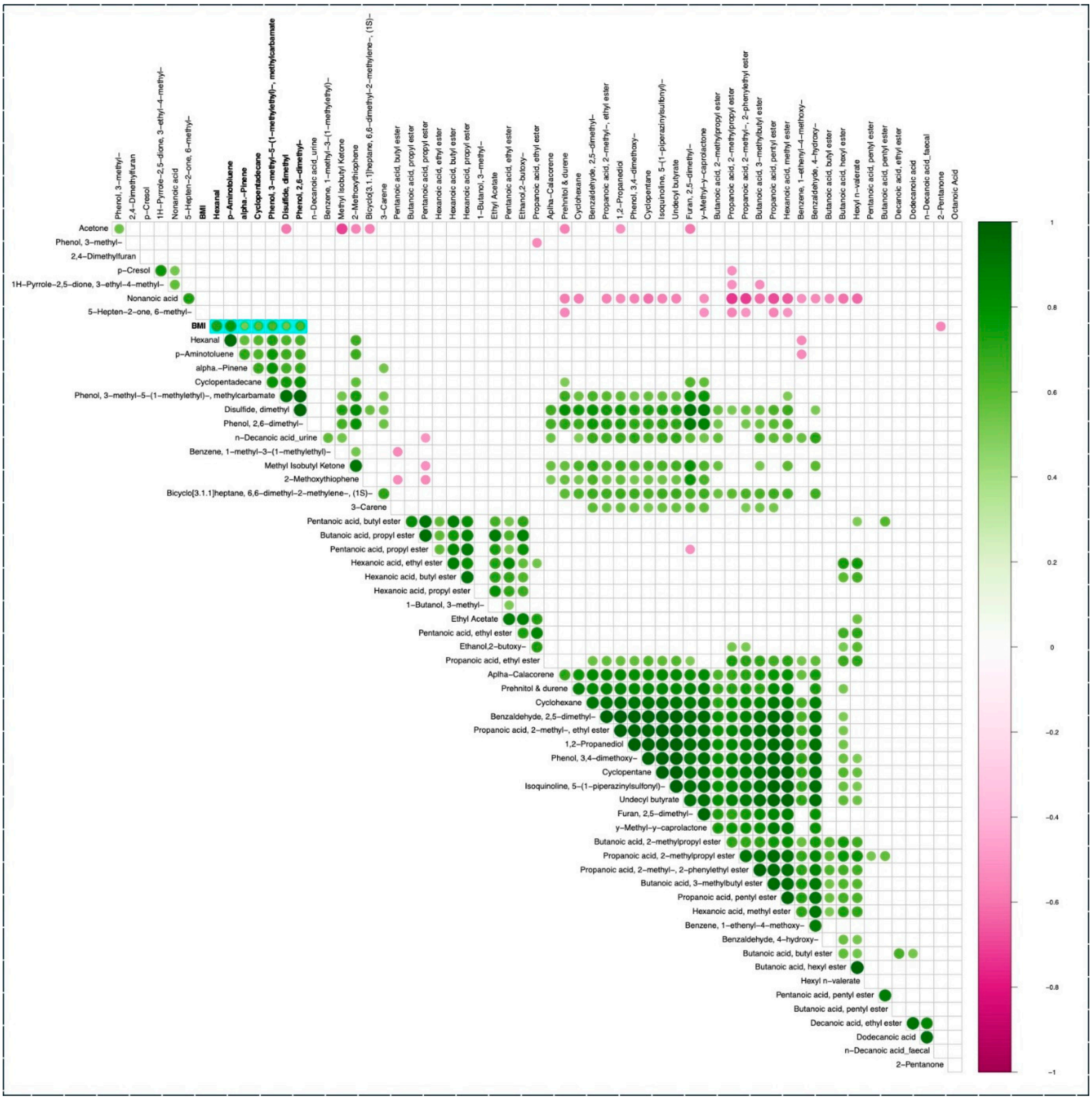

Supplementary Figure S3. Urinary indican (panel A) and urinary skatole (panel B) levels in patients with obesity before (T_0) and after (T_1) eight weeks of VLCKD. Data expressed as means ± SEM. Wilcoxon matched-pairs signed-rank test was used to compare pre-treatment and post-treatment data. Differences were considered significant at $p < 0.05$. The dotted line indicates the cut-off level for dysbiosis: indican (20 mg/L) and skatole (20 µg/L).

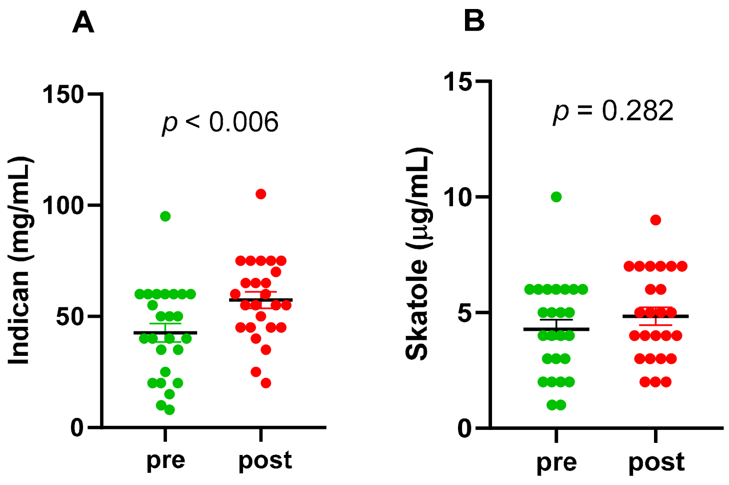

Supplementary Table S1. Wilcoxon rank sum test on faecal VOCs before and after the VLCKD. Statistically significant VOC emerging from the comparison between T0 and T1 groups have been reported together with their standard error of the mean (SEM). Underlined VOCs co-occurred in PLS-DA analysis.

**Statistically significant VOCs in faeces**	**Average (SEM) VLCKD - T0**	**Average (SEM) VLCKD - T1**	**VLCKD T0 Vs T1 (p value)**
Ethanol,2-butoxy-	1.36±0.449	0.55±0.433	0.0413
<u>alpha.-Pinene</u>	1.27±0.497	0.29±0.141	0.02299
1-Butanol, 3-methyl-	1.59±0.949	0.07±0.02	0.00658
<u>1,2-Propanediol</u>	0.04±0.01	0.08±0.016	0.00460
1H-Pyrrole-2,5-dione, 3-ethyl-4-methyl-	1.99±0.273	3.87±0.923	0.02748
5-Hepten-2-one, 6-methyl-	5.28±1.138	2.76±0.639	0.03668
Benzaldehyde, 4-hydroxy-	0.02±0.006	0.1±0.039	0.04456

Benzene, 1-methyl-3-(1-methylethyl)-	0.4±0.184	0.13±0.106	0.04258
Bicyclo[3.1.1]heptane, 6,6-dimethyl-2-methylene-, (1S)-	1.35±0.501	0.14±0.089	0.01286
Butanoic acid, 2-methylpropyl ester	0.42±0.246	0.02±0.014	0.00391
Butanoic acid, butyl ester	2.83±1.136	0.91±0.766	0.00053
Butanoic acid, hexyl ester	0.73±0.304	0.02±0.013	0.00391
Butanoic acid, propyl ester	3.49±1.272	1.83±1.624	0.04248
Cyclohexane	0.17±0.058	0.29±0.061	0.03607
Cyclopentadecane	0.28±0.181	1.05±0.549	0.01343
Cyclopentane	0.11±0.046	0.02±0.008	0.03906
Decanoic acid, ethyl ester	0.75±0.536	0.06±0.048	0.02734
Dodecanoic acid	1.86±0.851	0.28±0.102	0.00056
Ethyl Acetate	1.58±0.626	1.52±1.095	0.02899
Hexanoic acid, butyl ester	2.07±0.955	0.02±0.016	0.01172
Hexanoic acid, propyl ester	1.6±1.11	0.01±0.007	0.00195
Hexyl n-valerate	0.7±0.313	0±0.004	0.00391
Isoquinoline, 5-(1-piperazinylsulfonyl)-	0.03±0.011	0±0.003	0.00781
n-Decanoic acid	4.41±2.36	0.75±0.197	0.01504
Octanoic Acid	5.78±2.182	3.6±2.889	0.02027

p-Aminotoluene	0.52±0.167	0.26±0.132	0.02899
Pentanoic acid, pentyl ester	1.57±0.818	0.07±0.048	0.03125
Phenol, 3-methyl-5-(1-methylethyl)-, methylcarbamate	0.29±0.08	0.08±0.023	0.01273
Phenol, 3,4-dimethoxy-	0±0.002	0.06±0.04	0.02099
Propanoic acid, 2-methyl-, 2-phenylethyl ester	0.26±0.128	0.03±0.024	0.00085
Propanoic acid, 2-methyl-, ethyl ester	0.09±0.04	0.03±0.012	0.03015
Propanoic acid, 2-methylpropyl ester	0.6±0.228	0.14±0.111	0.00488
Propanoic acid, ethyl ester	1.13±0.339	0.79±0.442	0.01597
Propanoic acid, pentyl ester	0.35±0.128	0.01±0.009	0.00391
Undecyl butyrate	0.09±0.032	0±0.001	0.003916
Butanoic acid, 3-methylbutyl ester	0.3±0.12	0.01±0.004	0.01562
Hexanoic acid, ethyl ester	1.34±0.536	0.16±0.101	0.01660
Hexanoic acid, methyl ester	0.16±0.084	n.d.	0.03125
Pentanoic acid, ethyl ester	1.49±0.455	0.58±0.337	0.02496
3-Carene	1.47±0.56	0.37±0.23	0.01245
Butanoic acid, pentyl ester	1.73±0.737	0.18±0.159	0.00195
Pentanoic acid, butyl ester	2.73±1.046	0.27±0.147	0.00201

Pentanoic acid, propyl ester	2.45±0.99	0.28±0.246	0.02685
Phenol, 3-methyl	75.65±29.212	n.d.	0.01562

Supplementary Table S2. Wilcoxon rank sum test on urine VOCs before and after the VLCKD. Statistically significant VOCs have been listed based on statistically significant p-values. Standard error of the mean (SEM) has been reported for sample belonging to T0 and T1 groups. Underlined VOC are co-occurrent in PLS-DA analysis.

**Statistically significant VOCs in urines**	**Average (SEM) VLCKD - T0**	**Average (SEM) VLCKD - T1**	**VLCKD T0 Vs T1 (p value)**
Acetone	2.01±0.605	25.43±5.433	0.000003
Furan, 2,5-dimethyl-	0.36±0.04	0.24±0.032	0.003052
2-Pentanone	3.51±1.058	4.15±0.629	0.029106
2,4-Dimethylfuran	0.87±0.445	0.26±0.227	0.023438
Disulfide, dimethyl	0.51±0.056	0.33±0.044	0.018242
Methyl Isobutyl Ketone	1.59±0.102	1.08±0.1	0.002198
Hexanal	3±0.382	1.26±0.275	0.00025
2-Methoxythiophene	1.25±0.111	0.66±0.106	0.000056
Phenol, 2,6-dimethyl-	0.4±0.051	0.3±0.03	0.038599
Prehnitol & durene	0.5±0.055	0.68±0.083	0.030073
p-Cresol	3.77±0.455	6.36±1.232	0.029697
y-Methyl-y-caprolactone	0.18±0.042	0.25±0.031	0.042262
Benzene, 1-ethenyl-4-methoxy-	0.02±0.021	0.4±0.117	0.001953
Benzaldehyde, 2,5-dimethyl-	0.09±0.019	0.03±0.015	0.00534
Nonanoic acid	10.3±1.149	13.25±1.281	0.04215
n-Decanoic acid	0.44±0.12	0.76±0.153	0.028931
Aplha-Calacorene	0.44±0.146	1.06±0.253	0.020599

Supplementary Table S3. Pearson's correlation analysis between BMI and VOCs. If statistically significant, Pearson's correlation values and p-values relative to each correlation have been reported as bold font.

**VOC**	**correlation value against BMI**	**p-value**
Acetone	-0.016082126	0.939181555
Furan, 2,5-dimethyl-	0.358920916	0.078073049
**2-Pentanone**	**-0.546735197**	**0.004683707**

2,4-Dimethylfuran	0.269229487	0.19311611
**Disulfide, dimethyl**	**0.530633482**	**0.006354592**
Methyl Isobutyl Ketone	0.224796947	0.279989291
**Hexanal**	**0.713322244**	**6.26E-05**
2-Methoxythiophene	0.454060117	0.052607054
**Phenol, 2,6-dimethyl-**	**0.600186995**	**0.001515141**
Prehnitol & durene	0.156191172	0.455928413
p-Cresol	-0.261831474	0.206111728
y-Methyl-y-caprolactone	0.351761647	0.08464156
Benzene, 1-ethenyl-4-methoxy-	-0.3103245	0.131109168
Benzaldehyde, 2,5-dimethyl-	0.298099664	0.147802774
Nonanoic acid	-0.059076994	0.779088366
n-Decanoic acid_urine	0.095706452	0.649051466
Aplha-Calacorene	0.294787268	0.152575681
Ethanol,2-butoxy-	-0.039664639	0.85068581
**alpha.-Pinene**	**0.505486576**	**0.009947829**
1-Butanol, 3-methyl-	0.028185343	0.893609777
1,2-Propanediol	0.211504458	0.310142973
1H-Pyrrole-2,5-dione, 3-ethyl-4-methyl-	-0.304485666	0.138903241
5-Hepten-2-one, 6-methyl-	-0.301533038	0.142968882
Benzaldehyde, 4-hydroxy-	-0.071736638	0.733284721
Benzene, 1-methyl-3-(1-methylethyl)-	0.362828752	0.074655013
Bicyclo[3.1.1]heptane, 6,6-dimethyl-2-methylene-, (1S)-	0.028992453	0.890580853
Butanoic acid, 2-methylpropyl ester	0.105949829	0.614225831
Butanoic acid, butyl ester	-0.112012002	0.593985454
Butanoic acid, hexyl ester	-0.007361479	0.972140866
Butanoic acid, propyl ester	-0.11763174	0.575481203
Cyclohexane	0.116181797	0.580231074
**Cyclopentadecane**	**0.574791973**	**0.002652054**
Cyclopentane	0.220122934	0.290372401
Decanoic acid, ethyl ester	-0.00433944	0.983575585
Dodecanoic acid	-0.154260643	0.461584803
Ethyl Acetate	0.001948025	0.992626502
Hexanoic acid, butyl ester	-0.175829041	0.400507683
Hexanoic acid, propyl ester	-0.125657796	0.549504301
Hexyl n-valerate	-0.019648757	0.925726592
Isoquinoline, 5-(1-piperazinylsulfonyl)-	0.22001951	0.290604852
n-Decanoic acid_faecal	-0.187746695	0.368811044
Octanoic Acid	-0.119000959	0.571011582
**p-Aminotoluene**	**0.764986033**	**8.44E-06**
Pentanoic acid, pentyl ester	-0.073209797	0.728008544
**Phenol, 3-methyl-5-(1-methylethyl)-, methylcarbamate**	**0.611363094**	**0.001166701**

Phenol, 3,4-dimethoxy-	0.217155473	0.297088249
Propanoic acid, 2-methyl-, 2-phenylethyl ester	0.160659378	0.442977465
Propanoic acid, 2-methyl-, ethyl ester	0.256585656	0.215677834
Propanoic acid, 2-methylpropyl ester	0.10799199	0.607375797
Propanoic acid, ethyl ester	0.090019871	0.66870456
Propanoic acid, pentyl ester	0.165768907	0.428411546
Undecyl butyrate	0.215048479	0.301915039
Butanoic acid, 3-methylbutyl ester	0.163776472	0.434060265
Hexanoic acid, ethyl ester	-0.1084425	0.605868944
Hexanoic acid, methyl ester	0.187934674	0.368323079
Pentanoic acid, ethyl ester	0.08428066	0.68875664
3-Carene	0.234493116	0.259210081
Butanoic acid, pentyl ester	-0.157361249	0.452517922
Pentanoic acid, butyl ester	-0.230354499	0.267953821
Pentanoic acid, propyl ester	-0.187036158	0.370658875
Phenol, 3-methyl-	-0.449184834	0.064289451
BMI	1	0

- Bold font indicates statistically significant correlations.
